# Modulation of AKT Pathway-Targeting miRNAs for Cancer Cell Treatment with Natural Products

**DOI:** 10.3390/ijms24043688

**Published:** 2023-02-12

**Authors:** Jun-Ping Shiau, Ya-Ting Chuang, Ching-Yu Yen, Fang-Rong Chang, Kun-Han Yang, Ming-Feng Hou, Jen-Yang Tang, Hsueh-Wei Chang

**Affiliations:** 1 Division of Breast Oncology and Surgery, Department of Surgery, Kaohsiung Medical University Hospital, Kaohsiung Medical University, Kaohsiung 80708, Taiwan; 2 Graduate Institute of Medicine, College of Medicine, Kaohsiung Medical University, Kaohsiung 80708, Taiwan; 3 School of Dentistry, Taipei Medical University, Taipei 11031, Taiwan; 4 Department of Oral and Maxillofacial Surgery, Chi-Mei Medical Center, Tainan 71004, Taiwan; 5 Graduate Institute of Natural Products, Kaohsiung Medical University, Kaohsiung 80708, Taiwan; 6 Department of Biomedical Science and Environmental Biology, College of Life Science, Kaohsiung Medical University, Kaohsiung 80708, Taiwan; 7 School of Post-Baccalaureate Medicine, Kaohsiung Medical University, Kaohsiung 80708, Taiwan; 8 Department of Radiation Oncology, Kaohsiung Medical University Hospital, Kaohsiung Medical University, Kaohsiung 80708, Taiwan; 9 Institute of Medical Science and Technology, National Sun Yat-sen University, Kaohsiung 80424, Taiwan; 10 Center for Cancer Research, Kaohsiung Medical University, Kaohsiung 80708, Taiwan

**Keywords:** AKT, miRNA, bioactive substance, natural products, cell function

## Abstract

Many miRNAs are known to target the AKT serine-threonine kinase (AKT) pathway, which is critical for the regulation of several cell functions in cancer cell development. Many natural products exhibiting anticancer effects have been reported, but their connections to the AKT pathway (AKT and its effectors) and miRNAs have rarely been investigated. This review aimed to demarcate the relationship between miRNAs and the AKT pathway during the regulation of cancer cell functions by natural products. Identifying the connections between miRNAs and the AKT pathway and between miRNAs and natural products made it possible to establish an miRNA/AKT/natural product axis to facilitate a better understanding of their anticancer mechanisms. Moreover, the miRNA database (miRDB) was used to retrieve more AKT pathway-related target candidates for miRNAs. By evaluating the reported facts, the cell functions of these database-generated candidates were connected to natural products. Therefore, this review provides a comprehensive overview of the natural product/miRNA/AKT pathway in the modulation of cancer cell development.

## 1. Introduction

MicroRNAs (miRNAs) are part of a group of noncoding RNAs that exist in eukaryotic cells. They are approximately 21~22 nucleotides in length. miRNAs are responsible for post-transcriptional gene regulation and are classified according to their oncogenic and tumor suppression functions in the regulation of diverse cell functions [1]. miRNAs are known to have several functions in the regulation of proliferation, apoptosis [2], autophagy [3,4,5], endoplasmic reticulum (ER) stress [1,6], ferroptosis [7,8,9,10], necroptosis [11,12,13,14], DNA damage response (DDR) [15,16,17], senescence [18,19], and migration [20,21,22,23] relating to cancer cells [23,24,25]. In the following, we briefly introduce the background for these cell functions and their relationships to miRNA.

Apoptosis is a form of programmed cell death involving a series of activations for caspase signaling. Different miRNAs may exhibit functions opposite to apoptosis. Oncogenic miRNAs suppress cancer cell apoptosis, while tumor-suppressor miRNAs promote such apoptosis [2]. Autophagy refers to self-eating biomolecule recycling for energy restoration and abnormal organelle digestion of cells in response to starvation and other stressors. Autophagy-regulating miRNAs exhibit inhibitory or promoting effects in chemotherapy, radiotherapy, endocrine therapy, and target therapy for breast cancer [3]. Additionally, some autophagy-associated miRNAs contribute to modulating drug resistance to cancers [4,5].

ER stress occurs when protein misfolding exceeds the ER unfolding capacity. Unfolded protein response (UPR) is triggered by ER stress. When the ER stress is tolerable, cells adapt and can survive, but massive ER stress exceeding the unfolding capacity causes cell death. Oncogenic miRNAs promote early UPR and improve cancer cell survival but suppress late cell death response. In contrast, the effects of ER stress caused by tumor-suppressor miRNAs show the opposite function to that of oncogenic miRNAs [6]. Some miRNAs upregulate and downregulate ER stress in digestive cancer [1].

Ferroptosis refers to nonapoptosis-programmed cell death involving the upregulation of iron uptake, lipid peroxidation, and glutathione peroxidase 4 (GPX4) downregulation [26]. Ferroptosis-suppressing and -promoting miRNAs have been widely reviewed [7]. These miRNAs exhibit distinct functions targeting different components of ferroptosis signaling [8] and regulate ferroptosis among cancer cells [9,10]. Necroptosis refers to the nonapoptosis-programmed, necrosis-based cell death involving receptor-interacting serine/threonine-protein kinase 3 (RIPK3) and the mixed lineage kinase domain-like (MLKL) protein. Some miRNAs upregulate and downregulate necroptosis in the liver [11], kidneys [12], and lungs [13], as well as in colon cancer [14].

Canonical DDR includes the DNA damage sensors (MRE11/RAD50/NBS1 (MRN)), the *Ataxia telangiectasia* mutated (ATM) gene, the *Ataxia telangiectasia* and Rad3d-related (ATR) serine/threonine kinase, cell cycle checkpoints (checkpoint kinase 1/2 (CHK1/2) and p53), and downstream proapoptosis signaling [15]. Several miRNAs have been found to target DDR signaling, such as DNA damage sensing [15] and DNA repair [16,17]. Cellular senescence is characterized by permanent quiescent proliferation while maintaining metabolic activity. Senescent cells show a senescence-associated secretory phenotype (SASP) [18]. Modulating the SASP may control tumor growth. Several senescence-targeting miRNAs have been reviewed [18,19]. Moreover, migration and invasion are critical for metastases in cancer progression. Several miRNAs have been reported to regulate the detachment, epithelial-to-mesenchymal transition (EMT), migration, invasion, angiogenesis, and metastasis of cancer cells [20,21,22].

Notably, various miRNAs cooperate to control several cell functions. For example, apoptosis, necroptosis, and ferroptosis, which have close interactions, are mediated by reactive oxygen species (ROS) and lipid peroxidation [27]. Consequently, miRNAs regulating these cell functions may show complex interactions. However, the functions of miRNAs are commonly studied separately. Hence, detailed investigations into the combined interactions of miRNAs are warranted. This holds, for example, for the combined transfections of miR-424-5p and miR-142-3p, which cause separately greater antiproliferation, apoptosis, and autophagy in breast cancer cells than independent transfections [28]. Additionally, miR-34a-5p (miR-34a), miR-449a, and miR-16 are regarded as tumor-suppressor miRNAs. miR-34a and miR-16 are responsible for regulating senescence, autophagy, and apoptosis. miR-449a can control senescence and apoptosis but not autophagy. In cooperation with miR-16 and/or miR-34a-5p, miR-449a induces synergistic autophagy in cervical cancer cells [29]. Consequently, cell functions may be coordinately regulated by a network of miRNAs.

As mentioned, several miRNAs modulating cell functions have been reviewed in detail. However, the involvement of AKT serine-threonine kinase (AKT; protein kinase B (PKB)) and its effectors is less studied and the research lacks systemic organization. We introduce here the AKT (Section 2) and AKT effectors (Section 3) to connect the miRNA-mediated regulation of cell functions. The impacts of miRNAs (Section 4) and AKT- and AKT effector-targeting miRNAs (Section 5) on the treatment of cancer cells with natural products are particularly emphasized.

## *2.* Relationship between miRNA, AKT, and Cell Functions

Many types of cancer cells show high expression of AKT [30], which controls sophisticated cell functions (apoptosis, autophagy, ER stress, ferroptosis, necroptosis, DDR, senescence, and migration) [31,32,33,34] by cooperating with its downstream AKT effectors, such as forkhead box transcription factors (FOXO); c-Myc, the mechanistic target of rapamycin complex 1/2 (mTORC1/2); the mechanistic target of the rapamycin kinase (mTOR) substrate S6 kinase 1/2 (S6K1/2; PRS6KB1/2); sterol regulatory element-binding protein 1 (SREBP1); eukaryotic translation initiation factor 4E-binding protein 1 (4EBP1); and hypoxia-inducible factor (HIF) [33,34]. Furthermore, mTORC1 is composed of mTOR, RPTOR (regulatory-associated protein of mTOR complex 1; RAPTOR), mTOR-associated protein, LST8 homolog (MLST8), AKT1S1 (AKT1 substrate 1; PRAS40), and DEP domain-containing mTOR-interacting protein (DEPTOR).

**Figure 1:**
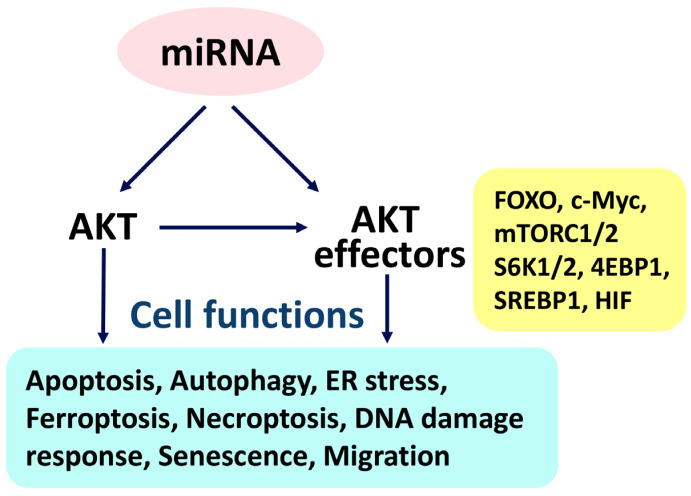
Overview of AKT, AKT effectors, and miRNAs and their regulation of multiple cell functions in cancer cells.

The various noncoding RNAs (ncRNAs), such as lncRNAs and circular RNAs, targeting AKT and its effectors in the regulation of a range of cell functions are well-reviewed [25,34]. However, the connections involving other ncRNAs, such as between miRNAs and AKT/AKT signaling, lack comprehensive and systemic integration. Recently, the impact of miRNAs in the regulation of AKT signaling has been reported in several cancer studies, such as for breast [35], glioblastoma [36], and nasopharyngeal [37] cancer cells. Moreover, miRNA and AKT signaling exhibit a reciprocal interaction in cancer cells [38]. Consequently, this review explored the relationship between miRNAs, AKT, and AKT effectors in connection to cell functions (Figure 1).

In the following (Section 2.1, Section 2.2, Section 2.3, Section 2.4, Section 2.5, Section 2.6, Section 2.7 and Section 2.8), AKT is connected to miRNA-mediated cell functions (Table 1).

### 2.1. miRNAs Targeting AKT-Mediated Pathways Regulate Apoptosis in Cancer Cells

Several miRNAs show apoptosis-modulating effects on cancer cells (Table 1). miR-330-3p (miR-330) shows lower expression in prostate cancer (PC-3) cells than normal cells. miR-330-3p can target several genes, such as E2F transcription factor 1 (*E2F1*) [39] and coatomer protein complex subunit beta2 (*COPB2*) [40], and regulate cancer cell proliferation. For example, miR-330-3p targets the 3′-untranslated region (3′-UTR) of *E2F1*. miR-330-3p triggers apoptosis of prostate cancer cells by downregulating E2F1 and AKT phosphorylation [39]. Furthermore, lysine demethylase 5A (KDM5A), a histone demethylase, is overexpressed in prostate cancer [40]. KDM5A silencing inhibits the proliferation and migration of prostate cancer cells. miR-330-3p targets the *COPB2* gene. KDM5A enhances prostate cancer (PRAD) cell proliferation by downregulating miR-330-3p and activating COPB2 and AKT expression [40].

**Table 1:** Connecting AKT to miRNA-regulated cell functions.

	Apoptosis	Autophagy	ER Stress	Ferroptosis	Necroptosis	DNA Damage Response	Senescence	Migration
**upregulation**	miR-330-3p [40] (↓AKT, prostate ca)	miR-21-5p [41] (↑AKT, glioma)				miR-374b [42] (↑AKT, colon ca)	miR-22-3p [43] (↓AKT3, endothelial cells)	miR-136 [44] (↑AKT, gastric ca)
	miR-641 [45] (↓AKT, breast ca)	miR-29a-3p [46] (↓AKT, osteosarcoma)				miR-22-3p [47] (↑AKT)	miR-181a-5p and miR-182-5p [48] (↓AKT3, uterine ca)	
	miR-107 [49] (↓AKT, hypopharyngeal ca)						miR-34a-5p [50] (↓AKT, chondrosarcoma)	
	miR-373-3p [51] (↓AKT, liver ca)						miR-145-5p [52] (vascular smooth muscle)	
	miR-1306-5p [53] (↓AKT, colon ca)							
	miR-181c [54] (↓AKT, resistant ovarian ca)							
**downregulation**	miR-14669 [55] (↓AKT, colon ca)		miR-495-3p [56] (↓AKT, breast ca)	miR-7-5p [57] (oral ca)	miR-26a-5p [58] (↑AKT, chicken kidney)			miR-495-3p [56] (↓AKT, breast ca)
	miR-126-3p [59] (↑AKT, lung ca)				miRNA-124-3p [60] (renal ca)			miR-200c-3p [61] (↓AKT, endometrial ca)
					miR-381-3p [62] (renal ca)			miR-148a [63] (↓AKT, liver ca)
					miR-29b-3p [64] (colon ca)			miR-107 [49] (↓AKT, hypopharyngeal ca)
								miR-373-3p [51] (↓AKT, liver ca)
								miR-1306-5p [53] (↓AKT, liver ca)

ca, cancer; ↑, enhance or activate; ↓, inhibit or inactivate. AKT or tissue information is not shown for some miRNAs because they were not provided in the literature collected by searching PubMed.

Several miRNAs are expressed at low levels in some cancer tissues. Overexpression of nonabundant miRNAs triggers apoptosis in cancer cells (Table 1). miR-641 is expressed at a low level and NUCKS1 shows a high level in breast cancer cells (Hs-578T, MCF7, HCC1937, and MAD-MB-231) [45]. Nuclear casein kinase and cyclin-dependent kinase substrate 1 (NUCKS1), a phosphoinositide 3-kinase (PI3K)/AKT enhancer, targets miR-641. miR-641 inhibits breast cancer cell migration and enhances apoptosis, which is reversed by PI3K inhibitor, suggesting miR-641-induced apoptosis through the targeting of the NUCKS1/PI3K/AKT axis [45]. miR-107 is downregulated in hypopharyngeal cancer compared to normal control tissues. miR-107 overexpression causes antiproliferative effects and apoptosis in hypopharyngeal cancer cells through the inactivation of PI3K/AKT [49]. miR-373-3p is downregulated [51] and transcription factor-activating enhancer-binding protein 4 (TFAP4) is upregulated [65] in liver cancer tissues. TFAP4 activates PI3K/AKT expression [65]. Upregulating miR-373-3p triggers apoptosis in liver cancer cells by downregulating AKT and TFAP4, which is reversed by TFAP4 overexpression [51]. miR-1306-5p is downregulated in colon cancer cells and tissues [53]. miR-1306-5p targets solute carrier organic anion transporter family member 2A1 (*SLCO2A1*). Overexpressing miR-1306-5p suppresses AKT signaling. Overexpressing miR-1306-5p inhibits proliferation and enhances apoptosis in colon cancer cells by inactivating AKT and targeting *SLCO2A1* [53].

Several miRNAs reduce drug resistance and induce apoptosis in cancer cells (Table 1). Paclitaxel is a first-line drug for ovarian cancer therapy, but its resistance problem limits its treatment effects [66]. miR-181c binds to the 3′-UTR of glucose-regulated protein 78 (GRP78) and downregulates its expression, suppressing the resistance of ovarian cancer (SKOV3-PTX) cells toward paclitaxel by inducing apoptosis [54]. miR-181c is downregulated in paclitaxel-resistant ovarian cancer cells. In contrast, upregulating miR-181c alleviates this paclitaxel resistance by inactivating AKT [54]. Therefore, miR-181c reduces the paclitaxel resistance involving AKT.

In contrast, some miRNAs inhibit apoptosis in cancer cells (Table 1). miR-126-3p (miR-126) silencing upregulates apoptosis gene expression and inhibits the proliferation of lung cancer cells by upregulating p-PI3K and p-mTOR expression [59]. Ginsenoside Rg1 shows antiproliferative and apoptotic effects in lung cancer (A540) cells by downregulating miR-126-3p [59]. This warrants a detailed assessment of the apoptotic role of miR-126-3p and AKT signaling in ginsenoside Rg1-treated lung cancer cells.

Other apoptosis-inhibitory miRNAs, such as miR-14669, enhance drug resistance (Table 1). Drug resistance in breast cancer can be increased due to the activation of the PI3K/AKT/mTOR axis [67]. miRNA-mediated apoptosis in colon cancer cells and PI3K/AKT signaling may partly contribute to drug resistance [55]. Vincristine, which inhibits miR-14669, induces apoptosis in colon cancer cells and alleviates drug resistance by inactivating PI3K/AKT signaling, which is reversed by the upregulation of miRNA-14669 [55]. As mentioned above, some miRNAs regulate apoptosis with the involvement of AKT.

### 2.2. miRNAs Targeting AKT Regulate Autophagy in Cancer Cells

Several miRNAs show autophagy-modulating effects in cancer cells (Table 1). miR-21-5p (miR-21) is an oncogenic miRNA in malignant glioma. miR-21-5p silencing by antisense oligonucleotide reduces radioresistance and AKT phosphorylation in γ-ray irradiated glioma cells, which is reversed by miR-21-5p overexpression [41]. Moreover, miR-21-5p silencing also induces autophagosome formation for autophagy. Hence, miR-21-5p stimulates AKT activation and suppresses autophagy, conferring radioresistance in glioma cells.

Some miRNAs show tumor-suppressive effects in cancer cells (Table 1). miR-29a-3p, a tumor-suppressive miRNA, exhibits low levels in osteosarcoma cells compared to normal cells [46]. Insulin-like growth factor 1 (*IGF1*) is a target of miR-29a-3p. IGF1 exerts oncogenic effects in osteosarcoma by inducing IGF-1R/PI3K/AKT activation, which is reversed by upregulating miR-29a-3p, triggering autophagy and antiproliferative effects [46]. Accordingly, some miRNAs regulate autophagy with the involvement of AKT.

### 2.3. miRNAs Targeting AKT Regulate ER Stress in Cancer Cells

Several miRNAs show ER stress-modulating effects in cancer cells (Table 1). miR-495-3p mimics suppress the expression of ER chaperone GRP78 and inhibit the proliferation and migration of breast cancer (MDA-MB-231) cells, reducing pirarubicin resistance by inactivating AKT expression, which is reversed by miR-495-3p inhibition [56]. Although studies on the targeting of ER stress-associated AKT by miRNAs are rare (Table 1), the literature on ER stress-associated AKT effectors targeted by various miRNAs is discussed later (Section 3.3).

### 2.4. miRNAs Targeting AKT Regulate Ferroptosis in Cancer Cells

Several miRNAs show ferroptosis-modulating effects in cancer cells (Table 1). Clinically relevant radioresistant oral cancer cells [68] exhibit miR-7-5p (miR-7) overexpression, which is reversed by miR-7-5p inhibition [57]. miR-7-5p silencing enhances ROS and intracellular Fe^2+^ content and upregulates ferroptosis gene (arachidonate 12-lipoxygenase, 12S type; ALOX12) expression and lipid peroxidation [57]. Although the reported study did not assess the involvement of AKT, miR-7-5p was predicted to target *AKT3* according to the miRDB database (retrieval date: 12 October 2022) [69]. This warrants a detailed evaluation of the role of miR-7-5p in regulating ferroptosis through the targeting of *AKT* in the future. Although studies on the targeting of ferroptosis-associated AKT by miRNA are rare (Table 1), the literature on ferroptosis-associated AKT effectors targeted by various miRNAs is discussed later (Section 3.4).

### 2.5. miRNAs Targeting AKT Regulate Necroptosis in Cancer Cells

Several miRNAs show necroptosis-modulating effects in noncancer cells (Table 1). Selenium yeast (Se-Y), an organic selenium source, suppresses cadmium-induced necroptosis in chicken kidney by downregulating RIP1, RIP3, and MLKL [58]. Moreover, Se-Y upregulates miR-26a-5p expression, causing PTEN downregulation and leading to AKT upregulation. Accordingly, miR-26a-5p may regulate necroptosis with the involvement of AKT.

Several miRNAs also show necroptosis-modulating effects in cancer cells (Table 1). miRNAs such as miRNA-124-3p (miRNA-124), miR-381-3p, and miR-29b-3p (miR-29b) are reported to show the ability to modulate necroptosis [62,64,69]. However, the role of AKT in this has not been investigated as yet. Cisplatin upregulates miRNA-124-3p in renal cancer (Caki-1) cells [60]. Calpain small subunit 1 (*CAPNS1*; *CAPN4*) is a target of miRNA-124-3p. Upregulating miRNA-124-3p alleviates cisplatin sensitivity, and cisplatin-induced necroptosis of renal cancer cells is reversed by CAPN4 overexpression [60]. miR-381-3p overexpression enhances cell proliferation and suppresses TNF-induced apoptosis and necroptosis of renal cancer cells [62]. Papillary renal cancer patients show high miR-381-3p expression and exhibit poor survival. Hence, miR-381-3p plays an oncogenic role in suppressing apoptosis and necroptosis. Additionally, miR-29b-3p is overexpressed, and TRAF5 is downregulated in colon cancer [64]. TNF receptor-associated factor 5 (*TRAF5*), a regulator of necroptosis, is a target of miR-29b-3p. Consequently, miR-29b-3p suppresses 5-fluorouracil-promoted necroptosis of colon cancer cells and enhances its resistance by downregulating TRAF5 [64].

Notably, after data mining using the miRDB database (retrieval date: 12 October 2022) [69], miRNA-124-3p and miR-29b-3p were predicted to target *AKT2* and *AKT*3, while miR-381-3p was predicted to target *AKT3*. Consequently, this warrants a careful examination of the role of AKT in miRNA-modulating (miRNA-124-3p, miR-381-3p, and miR-29b-3p) necroptosis in the future.

### 2.6. miRNAs Targeting AKT Regulate DDR in Cancer Cells

Several miRNAs show DDR-modulating effects, such as DNA damage in cancer cells (Table 1). Bleomycin causes DNA damage in colon cancer (HCT116 and HT29) cells accompanied by downregulation of AKT1 protein expression, which is reversed by p53 and miR-374b knockdown [42]. Overexpressing p53 promotes expression of the bleomycin-induced AKT1 regulator miR-374b, which is reversed by p53 knockdown. Hence, the p53/miR-374b/AKT1 axis regulates bleomycin-induced DNA damage in colon cancer cells.

In osteosarcoma cells, AKT1 and senescence suppress the DDR function, such as homologous recombination for DNA repair, of the mediator of DNA damage checkpoint 1 (MDC1) by overexpressing miR-22-3p (miR-22) [47]. Therefore, senescence downregulates MDC1 by upregulating miR-22-3p and downregulating DNA repair involving AKT activation. Accordingly, some miRNAs inhibit DDR with the involvement of AKT.

### 2.7. miRNAs Targeting AKT Regulate Senescence in Cancer Cells

Several miRNAs show senescence-promoting effects in cancer cells (Table 1). miR-22-3p is overexpressed in aged endothelial progenitor cells [43]. *AKT3* is a target of miR-22-3p. Upregulating miR-22-3p in young endothelial progenitor cells causes senescence and suppresses proliferation, migration, and angiogenesis, which is reversed by miR-22-3p silencing and AKT3 upregulation [43]. Hence, miR-22-3p regulates senescence with the involvement of AKT3. In addition to miR-22-3p, AKT3 is also regulated by other miRNAs. For example, the overexpression of miR-181a-5p (miR-181a) and miR-182-5p (miR-182) causes senescence in uterine leiomyoma cells by suppressing AKT3 and CCND2, respectively [48].

miR-34a-5p upregulation commonly occurs in the cartilage of osteoarthritis patients [50]. Delta-like protein 1 (*DLL1*) mRNA is a target of miR-34a-5p. DLL1 overexpression upregulates AKT phosphorylation (activation). Overexpressing miR-34a-5p has antiproliferative effects on and causes the senescence of chondrosarcoma cells by downregulating DLL1 and phosphorylated AKT, which is reversed by an miR-34a-5p inhibitor (Table 1) [50]. Hence, miR-34a-5p regulates senescence with the involvement of AKT. miR-145-5p (miR-145) overexpression is associated with DNA damage and senescence in vascular smooth muscle cells [52]. Moreover, miR-145-5p was predicted to target *AKT3* in accordance with the miRDB database (retrieval date: 12 October 2022) [69]. This warrants an advanced investigation of the AKT response in miR-145-5p-modulating senescence in the future. Although studies on the targeting of senescence-associated AKT by miRNA are rare (Table 1), the literature on senescence-associated AKT effectors targeted by several miRNAs is discussed later (Section 3.7).

### 2.8. miRNA Targeting AKT Regulates Migration in Cancer Cells

Several miRNAs function as tumor-suppressor miRNAs inhibiting cancer cell migration (Table 1). miR-200c-3p (miR-200c) suppresses EMT and dephosphorylates AKT in endometrial cancer cells by targeting the BMI1 proto-oncogene polycomb ring finger (*BMI-1*) gene, which is reversed by miR-200c inhibition [61]. miR-148a shows low expression in liver cancer tissues [63]. Death receptor-5 (*DR-5*) is a target of miR-148a. Overexpressing miR-148a suppresses proliferation, migration, and invasion and enhances apoptosis in liver cancer cells by downregulating EMT and AKT through the targeting of *DR-5* [63]. Upregulating oncogenic miR-107 inhibits the migration and invasion of hypopharyngeal cancer (FaDu) cells by inactivating AKT [49]. miR-373-3p functions as a tumor-suppressor miRNA and is generally downregulated in liver cancer cells. Overexpressing miR-373-3p inhibits liver cancer (Huh7, HLE, HCCLM6, and HCCLM3) cell migration, metastasis, and EMT by downregulating AKT activation [51]. miR-1306-5p, a tumor-suppressor miRNA, is expressed at a low level in colon cancer. Upregulating miR-1306-5p suppresses migration and invasion of colon cancer cells by inhibiting AKT activation [53].

In contrast, some miRNAs function as oncogenic miRNAs that inhibit cancer cell migration (Table 1). miR-136 is overexpressed in gastric cancer tissues and cells [44]. Downregulating miR-136 promotes antiproliferative effects and suppresses invasion in gastric cancer (MGC-803 and SGC-7901) cells. *PTEN*, a target of miR-136 and an inhibitor of AKT, shows low expression in gastric cancer tissues. miR-136 inhibition downregulates AKT phosphorylation [44]. Accordingly, some miRNAs regulate migration with the involvement of AKT.

## 3. Relationship between miRNA, AKT Effectors, and Cell Functions

In the following (Section 3.1, Section 3.2, Section 3.3, Section 3.4, Section 3.5, Section 3.6, Section 3.7 and Section 3.8), AKT effectors are connected to miRNA-mediated cell functions (Table 2).

### 3.1. miRNAs Targeting AKT Effectors Regulate Apoptosis in Cancer Cells

#### 3.1.1. FOXO-Targeting miRNAs and Apoptosis

FOXO plays a crucial role in promoting apoptosis by inducing proapoptotic protein expressions [70,71]. Several miRNAs regulate apoptosis in cancer cells with the involvement of FOXO (Table 2). miR-181a-5p is highly expressed in cervical cancer cells and is an oncogenic miRNA. Downregulating miR-181a-5p inhibits proliferation and invasion and triggers apoptosis in cervical cancer (HeLa) cells by upregulating PTEN and downregulating AKT and FOXO1 [72]. miR-335-5p (miR-335) shows a lower level in liver cancer cells than normal. miR-335-5p binds *FOXO3a* and targets *FOXO3a* 3′-UTR promoter activity. The miR-335-5p mimic enhances antiproliferative effects and apoptosis in liver cancer cells, which is reversed by the miR-335-5p inhibitor. Consequently, miR-335-5p triggers apoptosis in liver cancer (SMMC7721) cells by targeting *FOXO3a* [73].

**Table 2:** Connecting AKT effectors to miRNA-regulated cell functions.

AKT Effectors	Apoptosis	Autophagy	ER Stress	Ferroptosis	Necroptosis	DNA Damage Response	Senescence	Migration
**FOXO**	miR-181a-5p [72] (↑\↓), cervical ca	miR-223-3p [74] (↓\↓), liver ca	miR-132-3p [1] (↓\↓), colon ca	miR-670-3p [75] (↑\↓), liver ca	miR-6852-5p [76] (↑\↑), cervical ca	miR-223-3p [77] (↓\↓damage), breast ca	miR-34a-5p [78] (↑\↑), endothelial cells	miR-135a [79] (↓\↑), liver ca
	miR-335-5p [73] (↓\↑), liver ca		miR-494-3p [80] (↓FOXO3\↑)			miR-96 [81,82] ↓\↓repair), breast ca		
**c-Myc**	miR-196b-5p [83] (↓\↑), endometriotic stromal cells	miR-27b-3p [84] (↓\↓), resistant colon ca	miR-1291 [85] (↓\↓), prostate ca	miR-25-3p [86] (↓\↑), prostate ca	miR-494-3p [87] (↓\↓), ovarian ca	miR-1245 [88] (↑\↓repair), breast ca	miR-34a-5p [89] (↓\↑), liver ca	miR-33b-5p [90] (↓\↓), osteosarcoma
		miR-150-5p [91] (↑\↓), lung ca				miR-449a [92] (↓\+ damage), prostate ca		miR-34a-5p [93] (↓\↓), bladder ca
**mTORC1**	mi-21-5p [94] (↑mTOR\↓), renal cancer cells	miR-126-3p [95] (↓mTOR\↑), colon ca	miR-99b-5p and miR-100-5p [96] ↓mTOR\↑)	miR-137 [97] (↓mTOR\↓), melanoma	miR-32 [98,99] (↓mTOR\↓), epithelial	miR-18a-5p and miR-421-3p [100] (↑mTOR\↓)	miR-100-5p and miR-101-3p (↓mTOR\↑), endothelial cells	miR-520a-3p [101] (↓mTOR\↓), lung ca
	miR-1908 [102] ↓AKT1S1\↑), lung ca	miR-56 [103] (↓AKT1S1\↓), glioblastoma					miR-107 [104] (↑mTOR\↑), endothelial cells	miR-155-5p [105] (↓DEPTOR\↑), lymphoma
	miR-155-5p [105] (↑DEPTOR\↑), B-cell lymphoma	miR-125a-5p [106] (↓AKT1S1\↓), breast ca						
		miR-182-5p [107] (↓DEPTOR\↓), intestine I/R						
**S6K1/2**	X	miR-495-3p [108] (↑\↓), gastric ca	X	X	X	X	X	X
**SREBP1**	miR-185-5p and miR-342 [109] (↓\↑), prostate ca	miR-34a-5p [110] (↑\↑), fat liver						
	miR-132-3p [111] (↓\↑), glioma cells	miR-33 [112] (↑\↓), macrophages	miR-34a-5p [110] (↑\↑), fat liver	miR-670-3p [75] (↓\↓), liver ca	X	X	miR-21-5p [113] (↑\↓), prostate ca	miR-18a-5p [114] (↓\↓), breast ca
**4EBP1**	miR-149-3p [115] (↑\↓), T-ALL	miR-495-3p [108] (↑\↓), gastric ca	X	miR-1911-3p [116] (↓\↑), lung ca	X	X	miR-125a-5p and miR-125b-5p [117] (↓\↑), ovarian ca	miR-125a-5p and miR-125b-5p (↓\↓), ovarian ca
	miR-101-3p [118] (↓\↑), endometrial ca							
**HIF**	miR-199a-5p [119] (↓\↑), hemangioma-derived endothelial cells	miR-210 [120] (↑\↑), colon ca	miR-205-5p [121] (↑\↓), renal tubule cells	miR-147a [122] (↑\↑), glioblastoma	miR-210 (↑\↑ and miR-383 (↓\↓), [123] macrophage	miR-210 and miR-373 [124] (↑\↓), breast ca	miR-126-3p [125] (↑\↓), endothelial cells	miR-200c [126] (↓\↓), lung ca

ca, cancer; ↑, enhance or activate; ↓, inhibit or inactivate; left\right (e.g., ↓\↑ or ↑\↓), AKT effectors\cell function. mTORC1 is composed of mTOR, RPTOR, MLST8, AKT1S1, and DEPTOR. X indicates that miRNAs were rarely reported for these cell functions. X, data not available.

#### 3.1.2. c-Myc-Targeting miRNAs and Apoptosis

Several miRNAs regulate apoptosis in cancer cells with the involvement of c-Myc (Table 2). miR-196b-5p (miR-196b) overexpression suppresses proliferation and induces apoptosis in endometriotic stromal cells by targeting and downregulating c-Myc and Bcl-2 mRNA expression [83].

#### 3.1.3. mTOR-, AKT1S1-, and DEPTOR-Targeting miRNAs and Apoptosis

Several miRNAs regulate apoptosis in cancer cells with the involvement of MTORC1 (Table 2), which composed of mTOR, RPTOR, MLST8, and AKT1S1. miR-21-5p is overexpressed in renal cancer (ACHN) cells [94]. miR-21-5p silencing causes the apoptotic activation of caspase 3 and the dephosphorylation of signal transduction and activators of transcription 3 (STAT3). miR-21-5p enhances proliferation and suppresses apoptosis in renal cancer cells, which is reversed by mTOR inhibitor (XL388) [94].

The ribosomal protein-p53 pathway, a critical function for regulating apoptosis and senescence in cancer cells, is activated by the miR-1908 mimic and inhibited by AKT1S1 [127]. AKT1S1 downregulation promotes antiproliferative effects and apoptosis in melanoma (UACC 903) cells (Table 2) [102]. miR-1908 is downregulated in lung cancer cells. miR-1908 can target *AKT1S1* and downregulate ribosomal protein L11 (RPL11)-p53-p21 signaling [127]. Consequently, miR-1908 may induce apoptosis in lung cancer cells by targeting *AKT1S1*.

*DEPTOR* is a potential target of miR-155-5p (miR-155) [105], which regulates B-cell development, lymphomagenesis, and differentiation [128]. Inhibiting miR-155-5p enhances the sensitivity of ibrutinib-triggered apoptosis in diffuse large B-cell lymphoma cells to ibrutinib (Table 2) [105].

#### 3.1.4. SREBP1-Targeting miRNAs and Apoptosis

Several miRNAs regulate apoptosis in cancer cells with the involvement of SREBP1 (Table 2). SREBP1 is upregulated and miR-185-5p (miR-185) and miR-342-3p (miR-342) are downregulated in prostate cancer (LNCaP) cells compared to normal cells [109]. miR-185-5p and miR-342 suppress SREBP-1 and SREBP-2 expression, causing the inhibition of cell proliferation and migration in prostate cancer cells. Overexpression of miR-185-5p and miR-342-3p causes apoptosis in prostate cancer cells [109]. Additionally, miR-132-3p (miR-132) downregulates SREBP-1c expression; inhibits proliferation, migration, and invasion; and triggers apoptosis in glioma (U251) cells [111]. Hence, several miRNAs modulate SREBP1 expression in the regulation of apoptosis.

#### 3.1.5. EBP1-Targeting miRNAs and Apoptosis

Several miRNAs inhibit apoptosis in cancer cells with the involvement of 4EBP1 (Table 2). miR-149-3p (miR-149*) is overexpressed in T-cell acute lymphoblastic leukemia (T-ALL) [115]. miR-149-3p mimics enhance T-ALL cell proliferation and suppress apoptosis in T-ALL cells by upregulating 4EBP1 and S6K (PRS6KB1), which is reversed by miR-149-3p inhibitors [115]. In contrast, several miRNAs promote apoptosis in cancer cells with the involvement of SREBP1. miR-101-3p (miR-101) is downregulated and mTOR and 4EBP1 are upregulated in endometrial cancer cells, promoting proliferation and invasion and suppressing apoptosis, which is reversed by the miR-101-3p mimic and/or si-mTOR [118]. Hence, several miRNAs modulate 4EBP1 expression in the regulation of apoptosis.

#### 3.1.6. HIF1A-Targeting miRNAs and Apoptosis

Several miRNAs inhibit apoptosis in cancer cells with the involvement of HIF1A (Table 2). Proliferating hemangioma (HDEC) cells downregulate miR-199a-5p (miR-199a) and upregulate *HIF1A*, a target of miR-199a-5p [119]. The miR-199a-5p mimic suppresses the proliferation of hemangioma cells, which is reversed by HIF1A overexpression. The overexpression of miR-199a-5p causes antiproliferative effects and apoptosis in hemangioma-derived endothelial cells [119]. Hence, miRNA may regulate apoptosis with the involvement of HIF1A.

### 3.2. miRNAs Targeting AKT Effectors Regulate Autophagy in Cancer Cells

#### 3.2.1. FOXO- and c-Myc-Targeting miRNAs and Autophagy

Several miRNAs regulate apoptosis in cancer cells with the involvement of FOXO (Table 2). Doxorubicin downregulates miR-223-3p (miR-223) in liver cancer cells. FOXO3a is a direct target of miR-223-3p. miR-223-3p overexpression downregulates FOXO3a and suppresses doxorubicin-triggered autophagy, leading to chemoresistance [74].

Several miRNAs regulate apoptosis in cancer cells with the involvement of c-Myc (Table 2). After oxaliplatin treatment, resistant colon cancer cells show lower miR-27b-3p expression and higher autophagy than parental cells [84]. c-Myc downregulates miR-27b-3p expression. Hence, miR-27b-3p inhibits autophagy by downregulating c-Myc. Accordingly, c-Myc/miR-27b-3p signaling confers oxaliplatin resistance to colon cancer cells [84]. Furthermore, c-Myc also modulates other miRNAs. c-Myc and miR-150-5p (miR-150) are highly expressed in lung cancer (A549) cells [91]. c-Myc functions as a miR-150-5p transcriptional factor for upregulating miR-150-5p expression. miR-150-5p inhibits autophagy and enhances the proliferation of lung cancer cells. Consequently, the inhibition of c-Myc suppresses the proliferation of lung cancer cells.

#### 3.2.2. mTOR-, AKT1S1-, and DEPTOR-Targeting miRNAs and Autophagy

Several miRNAs induce the autophagy of cancer cells with the involvement of mTORC1 (Table 2). miR-126-3p shows a lower level in colon cancer tissues and cells than normal. miR-126-3p binds to 3′-UTR to downregulate mTOR expression. The overexpression of miR-126-3p inhibits proliferation and mTOR expression and induces autophagy and apoptosis in colon cancer cells, which is reversed by autophagy inhibitor bafilomycin A1, suggesting that miR-126-3p-induced apoptosis depends on autophagy through the modulation of mTOR expression [95].

In contrast, several miRNAs inhibit the autophagy of cancer cells with the involvement of mTORC1 (Table 2). miR-56 can directly target the 3′-UTR of *AKT1S1*. Overexpressing miR-56 enhances proliferation and suppresses autophagy in glioblastoma cells [103]. Hypermethylation of the CpG island located in the miR-125a-5p (miR-125a) promoter occurs in breast cancer tissues and cells, leading to miR-125a-5p downregulation, which is reversed by 5-Aza-dC, a methylation inhibitor. miR-125a-5p overexpression inhibits autophagy and enhances proliferation in breast cancer cells [106]. miR-125a-5p was predicted to target AKT effectors, such as *AKT1S1*, in accordance with the miRDB database [69] (retrieval date: 12 October 2022).

Intestinal ischemia/reperfusion (I/R) inhibits miR-182-5p expression. Overexpression of miR-182-5p inhibits autophagy and DEPTOR expression, alleviating intestinal damage under I/R (Table 2) [107]. This warrants an examination of DEPTOR in cancer cells.

#### 3.2.3. SREBP1-, 4EBP1-, S6K-, and HIF1A-Targeting miRNAs and Autophagy

Several miRNAs regulate the autophagy of cancer cells with the involvement of SREBP1 (Table 2). miR-33 has two isoforms, miR-33a-5p and miR-33b-5p (miR-33b), derived from intron 16 of SREBP2 and intron 17 of SREBP1 genes, respectively [129]. miR-33 can modulate the autophagy of macrophages by inhibiting several autophagy-related gene expressions [112]. In a high-fat-diet rat model, autophagy was induced in liver tissues by upregulating miR-34a-5p and SREBP1c [110].

Several miRNAs inhibit the autophagy of cancer cells with the involvement of 4EBP1 and S6K (Table 2). mTOR is an upstream regulator of autophagy that inhibits autophagosome formation. Multiple-drug-resistance (MDR) cells exhibit greater autophagy than parent cells. miR-495-3p upregulation suppresses autophagy in MDR gastric cancer (SGC7901) cells by phosphorylating mTOR substrates, such as 4EBP1 and PRS6KB1 (S6K1) [108]. Accordingly, miR-495-3p alleviates MDR by activating mTOR and inhibiting autophagy.

Under hypoxia, miR-210 is overexpressed and induces autophagy and radioresistance in colon cancer cells by upregulating HIF1A (Table 2) [120]. Hence, miRNA may regulate autophagy with the involvement of HIF1A. This warrants further identification of HIF1A-targeting miRNAs in the regulation of autophagy.

### 3.3. miRNAs Targeting AKT Effectors Regulate ER Stress in Cancer Cells

#### 3.3.1. FOXO-Targeting miRNAs and ER Stress

Several miRNAs control ER stress in digestive cancer with the involvement of FOXO (Table 2) [1]. miR-132-3p is overexpressed in gastric cancers [130]. Upregulated miR-132-3p promotes gastric cancer (AGS) cell growth by binding to the 3′-UTR of FOXO1 mRNA and, consequently, blocking FOXO1 function [130]. Folate depletion upregulates miR-132-3p and downregulates ER stress-associated gene expression in colon cancer cells [1]. Accordingly, FOXO1 is involved in miR-132-3p-regulated ER stress in cancer development.

Several studies have shown interactions between ER stress and miRNAs involving FOXO (Table 2). miR-494-3p is upregulated by ER stress inducers, such as tunicamycin and thapsigargin. miR-494-3p pretreatment suppresses tunicamycin-induced ER stress and promotes cell proliferation, which is reversed by miR-494-3p inhibition [80]. Hence, miR-494-3p shows reciprocal regulation with ER stress. Although this study did not assess the involvement of AKT effectors, miR-494-3p was predicted to target *FOXO3* in accordance with the miRDB database (retrieval date: 12 October 2022) [69]. This warrants a detailed evaluation of miR-494-3p in the regulation of ferroptosis through the targeting of AKT effectors in the future.

#### 3.3.2. c-Myc-Targeting miRNAs and ER Stress

Several miRNAs modulate ER stress in cancer cells with the involvement of c-Myc (Table 2). *IRE1α*, an ER stress sensor, is directly targeted by miR-1291 [85]. IRE1α enhances prostate tumor growth by activating c-Myc [131]. The inhibition of IRE1α downregulates c-Myc expression in natural killer cells. Accordingly, c-Myc is involved in miRNA-regulated ER stress.

#### 3.3.3. mTOR- and DEPTOR-Targeting miRNAs and ER Stress

Several miRNAs regulate ER stress in cancer cells with the involvement of mTORC1 (Table 2). *mTOR*, one of the targets of miR-99b-5p and miR-100-5p (miR-100), can trigger amyloid β-induced apoptosis by causing ER stress [96]. DEPTOR silencing causes multiple myeloma death without activating the UPR [132]. The role of DEPTOR in regulating ER stress in conjunction with miRNAs needs further investigation.

#### 3.3.4. SREBP1- and HIF1A-Targeting miRNAs and ER Stress

Several miRNAs regulate ER stress with the involvement of SREBP1 and HIF1A (Table 2). In a high-fat-diet rat model, liver miR-34a-5p was overexpressed and induced ER stress by downregulating sirtuin 1 (SIRT1), upregulating SREBP1c and GRP78 [110]. ER stress downregulates miR-205-5p (miR-205) and inhibits HIF1 expression in renal tubule cells [121]. This warrants a detailed investigation of SREBP1- and HIF1A-targeting miRNAs and their regulation of cancer cell autophagy.

### 3.4. miRNAs Targeting AKT Effectors Regulate Ferroptosis in Cancer Cells

#### 3.4.1. FOXO-, c-Myc-, and SREBP1-Targeting miRNAs and Ferroptosis

Several miRNAs modulate ferroptosis in cancer cells with the involvement of FOXO, c-Myc, and SREBP1 (Table 2). miR-670-3p, overexpressed in human glioblastoma [133], blocks ferroptosis by targeting acyl-CoA synthase long-chain family member 4 (*ACSL*4) [133]. Moreover, ACSL4 downregulates FOXO1 through ubiquitination and acetylation in β-cell-specific Rictor-knockout islets [75]. SREBP1 induces ACSL4 and lipogenesis-associated genes to improve the metastasis of liver cancer cells with c-Myc [134]. Additionally, the overexpression of lncRNA PCAT1 promotes docetaxel resistance and suppresses ferroptosis in prostate cancer (PC-3) cells by upregulating c-Myc and solute carrier family 7 member 11 (SLC7A11) [86]. SLC7A11 can compete with miR-25-3p and bind to c-Myc to enhance protein stability [86]. Accordingly, FOXO, c-Myc, and SREBP1 are involved in miRNA-regulated ferroptosis.

#### 3.4.2. mTOR-, 4EBP1-, and HIF1A-Targeting miRNAs and Ferroptosis

Several miRNAs modulate ferroptosis in cancer cells with the involvement of mTOR, 4EBP1, and HIF1A (Table 2). miR-137 inhibits ferroptosis by downregulating glutamine transporter SLC1A5 in melanoma (A375) cells [97]. SLC1A5 induces the uptake of glutamine and activates mTORC1 signaling [135]. Additionally, miR-1911-3p mimics downregulate mTORC1 enhancers, such as p-4EBP1, in lung cancer (H1299) cells [116]. Activated mTORC1 suppresses ferroptosis by upregulating 4EBP1 [136]. miR-147a, induced by HIF1A [137], binds to the 3′-UTR of solute carrier family 40 member 1 (*SLC40A1*) to trigger ferroptosis in glioblastoma (U87MG) cells [122]. Accordingly, mTOR, 4EBP1, and HIF1A are involved in miRNA-regulated ferroptosis.

### 3.5. miRNAs Targeting AKT Effectors Regulate Necroptosis in Cancer Cells

#### 3.5.1. FOXO- and c-Myc-Targeting miRNAs and Necroptosis

Several miRNAs modulate necroptosis in cancer cells with the involvement of FOXO and c-Myc (Table 2). Interleukin-27 upregulates miR-6852-5p and causes necrosis in cervical cancer cells by inhibiting Forkhead box protein M1 (FOXM1) expression [76], with the FOXM1 being suppressed by FOXO3a [138]. Upregulated miR-494-3p inhibits the expression of receptor-interacting serine/threonine-protein kinase 1 (RIPK1) in epilepsy rats [139], which is a necroptosis regulator [140]. Moreover, miR-494-3p targets the *c-Myc* and *SIRT1* genes in ovarian cancer (OVCAR3) cells [87]. Accordingly, FOXO and c-Myc are involved in miRNA-regulated necroptosis.

#### 3.5.2. mTOR- and HIF1A-Targeting miRNAs and Necroptosis

Several miRNAs modulate necroptosis is cancer cells with the involvement of mTOR and HIF1A (Table 2). Antibiotics decrease microbiota levels and downregulate the expression of receptor-interacting serine/threonine-protein kinase 3 (RIPK3), reducing mTOR hyperactivation-enhanced epithelial necroptosis [99]. Moreover, mTOR is downregulated by miR-32 [98]. Hence, the role of miR-32 in mTOR-regulated necroptosis warrants detailed assessments. Furthermore, miR-383 upregulates ATP contents and suppresses necroptosis. HIF1A triggers necroptosis and ATP depletion in inflammatory macrophages by upregulating miR-210 and downregulating miR-383 [123]. Accordingly, mTOR and HIF1A are involved in miRNA-regulated necroptosis.

### 3.6. miRNAs Targeting AKT Effectors Regulate DDR in Cancer Cells

#### 3.6.1. FOXO-Targeting miRNAs and DDR

Several miRNAs modulate DDR in cancer cells with the involvement of FOXO (Table 2). DNA damage agents, such as N-methyl-N’-nitro-N-nitrosoguanidine (MNNG), upregulate FOXO1 expression in lung cancer cells [141]. miR-223-3p downregulates FOXO1 to promote the proliferation of breast cancer, which is reversed by the miR-223-3p inhibitor [77]. Similarly, miR-96 inhibits FOXO3a expression in breast cancer cells through direct targeting [81]. FOXO3a exhibits a DNA repair function [82]. This warrants detailed assessments to examine the roles of miR-223-3p and miR-96 in FOXO-regulated DDR. Accordingly, FOXO is potentially involved in regulating miRNA-mediated DDR.

#### 3.6.2. c-Myc-Targeting miRNAs and DDR

Several miRNAs modulate DDR in cancer cells with the involvement of c-Myc (Table 2). c-Myc directly targets the miR-1245 promoter and enhances its expression, as well as causing downregulation of BRCA2 DNA repair-associated (*BRCA2)* gene expression and suppressing the ability for homologous recombination in breast cancer cells [88]. X-rays induce DNA damage, and they upregulate miR-449a but downregulate c-Myc in prostate cancer cells [92]. Accordingly, c-Myc is involved in regulating miRNA-mediated DDR.

#### 3.6.3. mTOR- and HIF1A-Targeting miRNAs and DDR

Several miRNAs modulate DDR in cancer cells with the involvement of mTOR and HIF1A (Table 2). mTORC1, a complex consisting of mTOR, MLST8, AKT1S1, and DEPTOR, activates S6K1 to inhibit ATM, a DNA damage sensor, by upregulating miR-18a-5p and miR-421-3p to target *ATM* [100]. In hypoxia, miR-210 and miR-373 are upregulated in HIF1A-positive breast cancer cells but not in HIF1A knockdown cells [124]. miR-210 overexpression downregulates the homologous recombination repair protein RAD52. miR-373 overexpression downregulates nucleotide excision repair (NER) proteins, such as RAD23B and RAD52 [124]. Consequently, miR-210 and miR-373 may function as oncogenic miRNAs by suppressing DNA repair.

### 3.7. miRNAs Targeting AKT Effectors Regulate Senescence in Cancer Cells

#### 3.7.1. FOXO-, c-Myc-, and mTOR-Targeting miRNAs and Senescence

Several miRNAs modulate senescence with the involvement of FOXO, c-Myc, and mTOR (Table 2). The overexpression of miR-34a-5p, a tumor-suppressor miRNA, inhibits SIRT1 and upregulates SIRT1 effector-acetylated FOXO1, suppressing angiogenesis by inducing the senescence of endothelial progenitor cells [78]. Furthermore, oncogene-induced senescence upregulates miR-34a-5p and downregulates c-Myc expression [89]. miR-34a-5p can bind to the 3′-UTR of c-Myc and FOXM1 and downregulate telomerase reverse transcriptase (hTERT) activity, causing senescence in liver cancer cells [89].

mTOR plays a vital role in miRNA-regulated senescence in endothelial cells [142]. miR-100-5p and miR-101-3p function as mTOR suppressors and modulate senescence [142]. Moreover, miR-107 can target *PTEN*, an MTORC1 suppressor, and upregulate its downstream factor MTORC1, leading to endothelial cell senescence (Table 2) [104].

#### 3.7.2. SREBP1-, 4EBP1-, and HIF1A-Targeting miRNAs and Senescence

Several miRNAs modulate senescence with the involvement of SREBP1, 4EBP1, and HIF1A (Table 2). miR-21-5p inhibition downregulates SREBP1 expression by suppressing insulin receptor substrate 1 (IRS1)-mediated transcription and inducing senescence in prostate cancer cells, which is reversed by miR-21-5p overexpression [113]. Moreover, 4EBP1 silencing triggers p53-dependent senescence [143]. The downregulation of 4EBP1 expression in ovarian cancer (SKOV3) cells causes the upregulation of miR-125a-5p and miR-125b-5p (miR-125b) [117]. *HIF1A* is a target of miR-126-3p [125]. Both miR-126-3p and HIF1A are downregulated in senescent endothelial cells. Consequently, SREBP1, 4EBP1, and HIF1A potentially regulate miRNA-mediated senescence.

### 3.8. miRNAs Targeting AKT Effectors Regulate Migration in Cancer Cells

#### 3.8.1. FOXO- and c-Myc-Targeting miRNAs and Migration

Several miRNAs modulate migration with the involvement of FOXO (Table 2). *FOXO1* is the direct target of miR-135a [79], which is overexpressed in liver cancer tissues and cells. Overexpression of miR-135a promotes the migration and invasion of liver cancer cells by upregulating MMP2 and downregulating FOXO3a, which is reversed by miR-135a inhibition [79]. Moreover, osteosarcoma tissues and cells exhibit low miR-33b-5p expression. miR-33b-5p inhibits osteosarcoma cell migration and invasion by downregulating c-Myc expression [90]. The *CD44* and *c-Myc* genes are the targets of miR-34a-5p. Overexpression of miR-34a-5p suppresses the invasion of bladder cancer (UMUC3) cells by downregulating CD44 and c-Myc [93]. Accordingly, FOXO and c-Myc are involved in regulating miRNA-mediated cancer cell migration.

#### 3.8.2. mTOR- and DEPTOR-Targeting miRNAs and Migration

Several miRNAs modulate migration with the involvement of mTOR and DEPTOR (Table 2). Overexpressing miR-520a-3p suppresses proliferation, migration, and invasion and triggers apoptosis in lung cancer (NCI-H1975) cells by inactivating AKT and downregulating mTOR, MMP2, and MMP9 [101]. miR-155-5p can directly bind to the 3′-UTR of DEPTOR to inhibit DEPTOR-mediated antimigration in diffuse large B-cell lymphoma cells [105]. Accordingly, MTORC1 is involved in regulating the miRNA-mediated migration of cancer cells.

#### 3.8.3. SREBP1-, 4EBP1-, and HIF1A-Targeting miRNAs and Migration

Several miRNAs modulate migration with the involvement of SREBP1 and 4EBP1 (Table 2). SREBP1, highly expressed in breast cancer, enhances proliferation and metastasis, contributing to the poor survival of breast cancer patients. SREBP1 is modulated by miRNAs, such as miR-18a-5p, which target *SREBP1* to suppress EMT status and invasion in breast cancer (MDA-MB-231) [114]. Additionally, miR-125a-5p and miR-125b-5p, potential tumor-suppressor miRNAs, are suppressed in ovarian cancer tissue and cells. The overexpression of miR-125a-5p and miR-125b-5p decreases the invasion and migration of ovarian cancer cells by downregulating 4EBP1 expression [117].

HIF1A can modulate several hypoxia-induced miRNAs (Table 2). miR-200c inhibits HIF1A expression and blocks the migration of lung cancer cells [126]. Therefore, overexpression of miR-200c may exhibit anticancer effects in tumors associated with hypoxia. Accordingly, SREBP1, 4EBP1, and HIF1A regulate the miRNA-mediated migration of cancer cells.

## 4. Modulation of miRNAs by Natural Products

Several natural products have been reported to modulate oncogenic and tumor-suppressor miRNAs and to control their targeting genes in the regulation of cancer cell proliferation [144,145,146]. Up-to-date, detailed information on the miRNAs, cancer cells, and functions related to these natural products is summarized in Table 3.

As shown in Table 3, ellagitannin, a fruit- and nut-derived polyphenol, has antiproliferative effects in liver cancer cells associated with miRNA modulation [147]. Ellagitannin upregulates 14 miRNAs (miR-526b-5p (miR-526b), miR-452-5p (miR-452), miR-194-5p (miR-194), miR-373-5p (miR-373*), miR-518-5p (miR-518f*), miR-302a-5p (miR-302a*), miR-424-5p (miR-424), let-7e-5p (let-7e), miR-525-5p (miR-525), miR-519e-5p (miR-519e*), miR-513a-3p (miR-513), miR-518c-5p (miR-518c*), miR-512-5p, and miR-346). It also downregulates seven miRNAs (miR-542-3p, let-7d-5p (let-7d), miR-299-3p, miR-200a-5p (miR-200a*), let-7f-5p (let-7f), let-7i-5p (let-7i), and let-7a-5p (let-7a)) [147].

The combined treatment including luteolin, ellagic acid, and punicic acid, which are pomegranate juice components, inhibits the metastasis of prostate cancer cells by enhancing the expression of tumor-suppressor miRNAs (miR-144-3p (miR-144), miR-133b, miR-1-3p (miR-1), miR-122-5p (miR-122), miR-34c-5p (miR-34c), miR-200c-3p, miR-127-3p (miR-127), miR-335-5p, miR-124-3p, miR-181a-5p, miR-7-5p, miR-15a-5p (miR-15a), and let-7d-5p). Moreover, this combined treatment also suppresses the expression of oncogenic miRNAs (miR-20a-5p (miR-20a), miR-21-5p, miR-9, miR-29b-3p, and miR-181b) [148] (Table 3).

A novel *Berberis amurensis* plant-derived berbamine analog, BBMD3, shows antiproliferative and apoptotic effects in glioblastoma cancer stem cells through the upregulation of miR-4284 [149]. Oridonin, a *Rabdosia rubescens*-derived diterpenoid, induces apoptosis in leukemia cells by suppressing miR-17-5p (miR-17) and miR-20a-5p [150]. Bufalin, a Chinese toad venom-derived toxin, suppresses differentiation and proliferation and triggers apoptosis in osteosarcoma cancer stem cells by upregulating miR-148a-3p [151]. Betulinic acid, a pentacyclic triterpenoid, suppresses liver cancer cell proliferation and enhances apoptosis by upregulating miR-21-5p [152] and miR-22-3p [153]. Piperlongumine, a *Piper longum*-derived alkaloid, causes ROS generation and apoptosis and downregulates c-Myc and its downstream miRNAs, such as miR-27a-3p (miR-27a), miR-20a-5p, and miR-17-5p, in pancreas, lung, and breast cancer cells [154] (Table 3).

Fucoidan has antiproliferative, apoptotic, and antimigration effects in liver cancer cells, accompanied by the downregulation of miR-522-3p [155]. Steviol, a *Stevia rebaudiana* Bertoni leaf-derived natural sweetener, shows anticancer effects, such as antiproliferative effects, G1 arrest, and apoptotic effects, in gastrointestinal cancer cells, accompanied by upregulation of miR-203a-3p and downregulation of miR-6088 in colon cancer (Caco-2) cells and upregulation of miR-1268b and downregulation of miR-23c in gastric cancer (HGC-27) cells [156]. The miRNAs involved in the anticancer effects of apigenin are well-reviewed [157]. For example, miR-101-3p (miR-101) was downregulated in doxorubicin-treated liver cancer cells, which was reversed by apigenin for apoptosis-inducible effects [158]. The Kanglaite Injection (KLT), made from the TCM herb yiyiren (Coicis Semen) through supercritical extraction [159], shows anticancer effects in lung cancer patients through the downregulation of miR-21-5p [160] (Table 3).

The natural product butylcycloheptyl prodiginine binds to precursor miR-21-5p to block the Dicer-mediated maturation of oncogenic miR-21-5p, inhibiting the growth of colon cancer cells [161]. Cucurbitacin B, a Cucurbitaceae plant-derived natural product, causes G2/M arrest and inhibits the proliferation of pancreatic cancer cells and xenograft tumors by upregulating miR-146b-5p [162]. Sanguinarine, an argemone oil-derived alkaloid, induces G1 arrest and oxidative stress-dependent apoptosis in liver cancer cells by upregulating miR-16-2 expression [163]. A methoxylated quercetin glycoside (quercetin-3′-methoxy-3-*O*-(4”-acetylrhamnoside)-7-*O*-α-rhamnoside) suppresses the proliferation and migration of liver cancer cells by upregulating TP53 and its downstream miRNAs miR-15a-5p (miR-15a)/miR-16-5p (miR-16), which can be reversed by inhibitors of miR-15a-5p/miR-16-5p (Table 3) [164].

Honokiol, a *Magnolia officinalis*-derived polyphenol, has antiproliferative and apoptotic effects in osteosarcoma cells through the downregulation of miR-21-5p (miR-21) [165]. miR-21-5p downregulates the phosphatase and tensin homolog (PTEN) [165], an AKT inhibitor. Consequently, honokiol inhibits miR-21-5p expression, causing AKT inactivation, which is reversed by miR-21-5p overexpression. Moreover, doxorubicin resistance develops from miR-188-5p overexpression, and honokiol can downregulate miR-188-5p to improve the doxorubicin sensitivity in breast cancer (MDA-MB-231) cells [166]. Shikonin, a *Lithospermum erythrorhizon* root-derived naphthoquinine, induces cell autophagy by upregulating miR-545-3p in colon cancer cells [167] (Table 3).

Sulforaphane, a cruciferous vegetable-derived sulfur-rich natural product, triggers cervical cancer cell apoptosis by activating MAPK through the downregulation of miR-1247-3p [168]. Sulforaphane also exhibits antiproliferative and apoptotic effects in nasopharyngeal cancer cells by increasing miR-124-3p expression [169]. Luteolin (3′,4′,5,7-tetrahydroxy flavone), a naturally occurring flavonoid modulating several miRNAs and inducing apoptosis in various types of cancer cells, is well-reviewed [170]. For example, miR-7-1-3p, miR-124-3p, miR-8080, miR-34a-5p, miR-384, miR-6809-5p, and miR-630 are upregulated by luteolin and exhibit tumor-suppressor functions in glioblastoma, brain, prostate, colon, gastric, liver, and prostate cancer cells, respectively [170] (Table 3).

Camptothecin, a *Camptotheca acuminate*-derived alkaloid, suppresses the migration, invasion, and proliferation of liver cancer Huh7 cells by upregulating miR-16-5p [171]. Piceatannol, a resveratrol analog, shows antiproliferative and apoptotic effects in osteosarcoma cells through the downregulation of miR-21-5p [172]. Matrine, a *Sophora flavescens* Ait-derived bioactive compound, triggers apoptosis in thyroid cancer (PTC) cells and inhibits tumor growth by decreasing miR-182-5p [173]. Maytenin and 22-β-hydroxymaytenin, which are *Maytenus ilicifolia* root-derived quinone-methide triterpenes, trigger ROS generation and apoptosis in head and neck cancer cells by downregulating miR-27a-3p, miR-20a-5p, and miR-17-5p [174]. Dihydromyricetin, an *Ampelopsis* plant-derived flavonoid, suppresses proliferation and migration, triggering cholangiocarcinoma cell apoptosis by downregulating miR-21-5p [175] (Table 3).

**Table 3:** Connecting natural products to miRNA-regulated cell functions.

Natural Products	miRNAs	Cancer	Cell Functions
Ellagitannin	↑ miR-526b-5p, miR-452-5p, miR-194-5p, miR-373-5p, miR-518-5p, miR-302a-5p, miR-424-5p, let-7e-5p, miR-525-5p, miR-519e-5p, miR-513a-3p, miR-518c-5p, miR-512-5p, miR-346 [147]	Liver ca	Antiproliferation
	↓ miR-542-3p, let-7d-5p, miR-299-3p, miR-200a-5p, let-7f-5p, let-7i-5p, let-7a-5p [147]		
Luteolin, ellagic acid, punicic acid (combined)	↑ miR-144-3p, miR-133b, miR-1-3p, miR-122-5p, miR-34c-5p, miR-200c-3p, miR-127-3p, miR-335-5p, miR-124-3p, miR-181a-5p, miR-7-5p, miR-15a-5p, let-7d-5p [148]	Prostate ca	Antimetastasis
	↓ miR-20a-5p, miR-21-5p, miR-9, miR-29b-3p, miR-181b [148]		
Berbamine analog BBMD3	↑ miR-4284 [149]	Glioblastoma	Antiproliferation, apoptosis
Oridonin	↓ miR-17-5p, miR-20a-5p [150]	Leukemia	Apoptosis
Bufalin	↑ miR-148a-3p [151]	Osteosarcoma	Antiproliferation, apoptosis
Betulinic acid	↑ miR-21-5p [152], miR-22-3p [153]	Liver ca	Antiproliferation, apoptosis
Piperlongumine	↓ miR-27a-3p, miR-20a-5p, miR-17-5p [154]	Pancreas, lung, breast ca	Apoptosis
Fucoidan	↑ miR-29b-3p [176]	Liver ca	Antimigration
	↓ miR-522-3p [155]	Liver ca	Antiproliferation, apoptosis, antimigration
Steviol	↑ miR-203a-3p; ↓ miR-6088 [156]	Colon ca	Antiproliferation, apoptosis
	↑ miR-1268b; ↓ miR-23c [156]	Gastric ca	Antiproliferation, apoptosis
Baicalein	↑ miR-3127-5p [177]	Liver ca	Anti-apoptosis
Apigenin	↑ miR-101-3p [158]	Liver ca	Apoptosis
Kanglaite injection	↓ miR-21-5p [160]	Lung ca patients	Anticancer
Butylcycloheptyl prodiginine	↓ miR-21-5p [161]	Colon ca	Antiproliferation
Cucurbitacin B	↑ miR-146b-5p [162]	Pancreatic ca	Antiproliferation
Sanguinarine	↑ miR-16-2 expression [163]	Liver ca	Apoptosis
Quercetin-3′-methoxy-3-O-(4″-acetylrhamnoside)-7-O-α-rhamnoside	↑ miR-15a-5p, miR-16-5p [164]	Liver ca	Antimigration
Honokiol	↓ miR-21-5p (miR-21) [165]	Osteosarcoma	Apoptosis
	↓ miR-188-5p [166]	Breast ca	Doxorubicin sensitization
Shikonin	↑ miR-545-3p [167]	Colon ca	Autophagy
Sulforaphane	↓ miR-1247-3p [168]	Cervical ca	Apoptosis
	↑ miR-124-3p expression [169]	Nasopharyngeal ca	Apoptosis
Luteolin	↑ miR-7-1-3p, miR-124-3p, miR-8080, miR-34a-5p, miR-384, miR-6809-5p, miR-630 [170]	Glioblastoma, brain, prostate, colon, gastric, liver, prostate ca	Apoptosis
Camptothecin	↑ miR-16-5p [171]	Liver ca	Antimigration
Piceatannol	↓ miR-21-5p [172]	Osteosarcoma	Apoptosis
*Panax ginseng* CA Meyer	↑ miR-21-5p [178]	Gastric precancerous lesion	Apoptosis
Oleacein	↑ miR-193a-3p, miR-193a-5p, miR-34a-5p, miR-16-5p [179]	Melanoma	Apoptosis
	↓ miR-214-3p [179]	Melanoma	Apoptosis
Matrine	↓ miR-182-5p [173]	Thyroid ca	Apoptosis
Maytenin, 22-β-hydroxymaytenin	↓ miR-27a-3p, miR-20a-5p, miR-17-5p [174]	Head/neck ca	Apoptosis
Dihydromyricetin	↓ miR-21-5p [175]	Cholangiocarcinoma	Antiproliferation, apoptosis
Toosendanin	↑ miR-608 [180]	Glioma	Apoptosis
(−)-Sativan	↑ miR-200c-3p [181]	Breast ca	Antimigration, apoptosis
Thymoquinone	↑ miR-34a-5p [182]	Breast ca	Antimigration
Phytic acid	↓ miR-224-5p, miR-200a-3p [183]	Colon ca	Apoptosis
Polyphenol-enriched blueberry preparation	↑ miR-200b-3p [184]	Melanoma	Antimigration
Formononetin	↓ miR-542-5p [185]	Gastric ca	Antimigration
Rutin	↑ miR-590-5p [186]	Liver ca	Anti-autophagy
	↑ miR-129-1-3p [187]	Breast ca	Antimetastasis
Asparanin A	↓ miR-551a, miR-1303 [188]	Endometrial ca	Antimigration
CoB1	↑ miR-125b-5p [189]	Lung ca	Autophagy
Curcumenol	↑ miR-19b-3p [190]	Lung ca	Ferroptosis
Hesperidin, luteolin	↑ miR-16-5p, miR-34a-5p [191]	Breast ca	Apoptosis
	↓ miR-21-5p [191]	Breast ca	Apoptosis
Sulforaphane/peptide nucleic acid (combined)	↓ miR-15b-5p [192]	Glioblastoma	Apoptosis
Solamargine	↑ miR-192-5p [193]	Liver ca	Apoptosis, autophagy
Asiaticoside	↑ miR-635 [194]	Gastric ca	ER stress
Icariside II	↑ miR-324-3p [195]	Renal ca	Ferroptosis

↑, enhance or activate; ↓, inhibit or inactivate.

Toosendanin, a *Melia toosendan* Sieb et Zucc-derived triterpenoid, promotes apoptosis in glioma cells by upregulating miR-608 [180]. (−)-Sativan, a *Spatholobus suberectus*-derived natural product, inhibits proliferation and migration and induces apoptosis in breast cancer cells by upregulating miR-200c-3p [181]. Thymoquinone, the main bioactive compound derived from *Nigella sativa*, inhibits the migration of breast cancer cells by upregulating miR-34a-5p [182]. Thymoquinone induces apoptosis by downregulating the activation (phosphorylation) of STAT3 [196]. Moreover, thymoquinone has chemosensitization effects. The combined treatment including thymoquinone and doxorubicin shows synergistic antiproliferative effects against murine solid Ehrlich carcinoma through the upregulation of miR-125a-5p [196]. Phytic acid, a plant seed-derived natural product, improved the apoptotic effects of oxaliplatin in a 1,2-dimethylhydrazine-induced colorectal cancer model by downregulating miR-224-5p (miR-224) and miR-200a-3p (miR-200a) [183] (Table 3).

The polyphenol-enriched blueberry preparation inhibits the proliferation and migration of melanoma cells by upregulating miR-200b-3p (miR-200b) [184]. Formononetin, an isoflavone derived from *Astragalus membranaceus* root, suppresses the migration and invasion of gastric cancer cells by downregulating miR-542-5p [185]. Rutin, a vegetable- and fruit-derived flavonoid, inhibits autophagy in liver cancer cells by downregulating the lncRNA BRAF-activated ncRNA (BANCR), a molecular sponge of miR-590-5p [186]. Accordingly, rutin upregulates miR-590-5p in liver cancer cells. Moreover, rutin also inhibits breast cancer cell proliferation and metastasis by upregulating miR-129-1-3p-dependent calcium signaling [187]. Asparanin A, an *Asparagus officinalis*-derived natural product, inhibits the migration and invasion of endometrial cancer cells by downregulating miR-551a and miR-1303 [188] (Table 3).

Curcumenol is a *Curcuma zedoaria*-derived sesquiterpene causing ferroptosis in lung cancer (H1299) cells by downregulating lncRNA H19 and upregulating miR-19b-3p [190]. Hesperidin and luteolin, both citrus fruit-derived flavanone glycosides and *Reseda luteola*-derived flavonoids, induce antiproliferative and apoptotic effects in breast cancer (MCF7) cells by downregulating miR-21-5p and upregulating miR-16-5p and miR-34a-5p [191]. A combined treatment including sulforaphane and peptide nucleic acid triggers the synergistic apoptosis of glioblastoma (U251) cells by downregulating miR-15b-5p [192]. Asiaticoside, a glycosylated triterpene of *Centella asiatica*, has antiproliferative effects and causes ER stress in gastric cancer cells by upregulating miR-635 [194]. Icariside II, the *Epimedium brevicornum*-derived natural product, induces ferroptosis in renal cancer cells by upregulating miR-324-3p to target *GPX4* [195]. Fucoidan, a sulfated brown algal polysaccharide, suppresses EMT in liver cancer (HepG2) cells by upregulating miR-29b-3p to inhibit DNMT3B, a metastasis suppressor 1 (MTSS1) suppressor [176] (Table 3).

**Figure 2:**
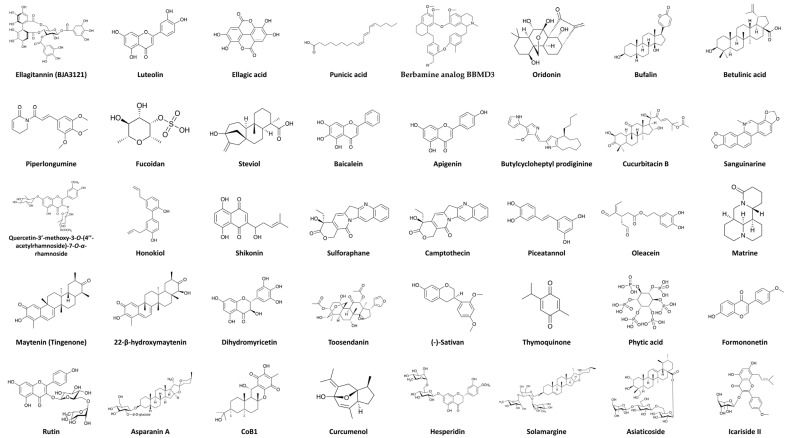
Chemical structures of the natural products mentioned in Table 3.

All chemical structures mentioned in Table 3 are provided in Figure 2.

## 5. Modulation of AKT- and AKT Effector-Targeting miRNAs by Natural Products

Many natural products show AKT- and AKT effector-modulating functions [197] with anticancer effects. However, a systemic understanding of the potential roles of miRNAs in regulating cell functions in coordination with natural products that modulate AKT and AKT effectors has not yet been achieved.

Several natural products that control various cancer cell functions by modulating miRNAs are summarized in Table 3. Some anticancer studies on natural products have emphasized the regulation of AKT and several AKT effectors in the miRNA-mediated cell functions exerted by natural products (Table 3). For example, solamargine, a *Solanum incanum* herb-derived natural product, promotes antiproliferative effects, apoptosis, and autophagy in liver cancer cells by downregulating leukemia inhibitory factor (LIF), cysteine-rich angiogenic inducer 61 (CYR61), and AKT and upregulating miR-192-5p (Table 3) [193]. Moreover, the downregulation of miR-192-5p enhances AKT activation. miR-3127-5p is downregulated to promote cell proliferation in several liver cancer cells. Baicalein can upregulate miR-3127-5p to suppress liver cancer (Bel-7402) cell proliferation, S phase arrest, and apoptosis by inactivating AKT (Table 3) [177]. *Panax ginseng* CA Meyer (Rg3) suppresses proliferation and promotes apoptosis in gastric precancerous lesion cells by upregulating miR-21-5p and downregulating PI3K/AKT (Table 3) [178]. Oleacein, an extra-virgin olive oil polyphenol, induces antiproliferative and apoptotic effects in melanoma cells by upregulating miR-193a-3p, miR-193a-5p (targeting *mTOR*), miR-34a-5p, and miR-16-5p (targeting *mTOR*) and downregulating miR-214-3p (targeting *BAX*) (Table 3) [179]. The cochlioquinone derivative CoB1 triggers autophagy in lung cancer (A549) cells by upregulating miR-125b-5p expression and downregulating AKT and FOXO3 expressions [189] (Table 3). Although these studies provide information on AKT and several AKT effectors, the potential functions of other AKT effectors in natural product-induced miRNA modulation remain unclear.

Notably, most studies listed in Table 3 did not investigate the involvement of AKT and its effectors. Utilizing bioinformatics (the miRDB database [69]), the miRNAs listed in Table 3 were input into the miRDB database to check the predicted target genes related to AKT and AKT effectors. The retrieval results for the natural product-modulating miRNAs from Table 3 are summarized in Table 4. For example, miR-7-1-3p was predicted to target *AKT1*, while miR-103a-3p, miR-107, miR-124-3p, miR-148a-3p, miR-29b-3p, and miR-29c-3p were predicted to target *AKT2* (Table 4). Many miRNAs were predicted to target *AKT3*, as shown in Table 4.

Many AKT effectors were predicted to be targeted by several natural product-modulated miRNAs. For example, *FOXO1* was predicted to be targeted by let-ff-1-3p, miR-143-3p, miR-144-3p, miR-145-5p, miR-182-5p, miR-183-5p, miR-223-3p, miR-27a-3p, miR-27b-3p, miR-324-5p, miR-486-5p, miR-513a-3p, miR-5195-3p, miR-7-1-3p, and miR-96-5p (Table 4). Similarly, *FOXO3* and *FOXO4* were predicted to be targeted by many of the natural product-modulated miRNAs from Table 3. *Myc* was predicted to be targeted by miR-16-2, miR-203a-3p, and miR-629-5p. *mTOR* was predicted to be targeted by miR-101-3p, miR-144-3p, miR-421, miR-545-3p, miR-616-3p, miR-629-5p, miR-96-5p, and miR-99a-5p. *AKT1S1* was predicted to be targeted by miR-124-3p, miR-125a-5p, miR-125b-5p, and miR-129-1-3p.

Moreover, *DEPTOR* and *HIF1A* were also predicted to be targeted by several natural product-modulating miRNAs that were not reported to be associated with AKT and AKT effectors. Therefore, several miRNAs connected to natural product studies have bioinformatic predictions related to AKT and AKT effectors that should be verified in experiments. The contributions of AKT and AKT effectors to the modulation of miRNAs by natural products can thus be explored.

## 6. Conclusions

Many miRNAs regulate various cancer cell functions by targeting several genes. AKT and AKT effectors are highly expressed in various cancer cells and accompanied by the regulation of cell functions (apoptosis, autophagy, ER stress, ferroptosis, necroptosis, DDR, senescence, and migration). However, the connection between miRNAs and AKT and AKT effectors in controlling cell function remains unclear. Although several natural products may modulate miRNAs and the AKT pathway, their relationships lack systematic organization.

**Figure 3:**
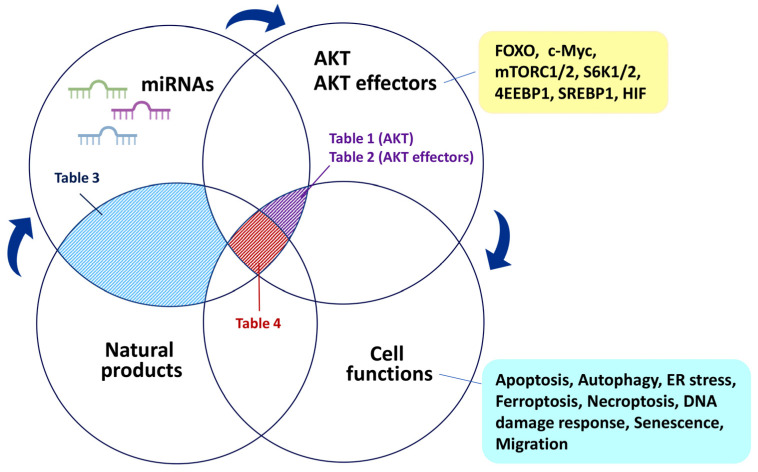
Schematic diagram of the natural products affecting the regulation of miRNAs that target AKT and AKT effectors to regulate multiple cell functions in cancer cells. More detailed information is provided in each of the tables above.

This review provided comprehensive information concerning the relationships between miRNAs and cancer cell functions (Figure 3). The roles of AKT and AKT effectors in miRNA-regulated cancer cell functions were clarified. Moreover, there is abundant information on the modulation of many miRNAs by natural products, but the involvement of AKT and AKT effectors has rarely been reported. Utilizing bioinformatics, the miRDB database was chosen for the prediction of AKT and AKT effectors related to miRNAs regulated by natural products. Consequently, the gaps between AKT, AKT effectors, miRNAs, and natural products were filled (Figure 3).

However, the predictions of the targets for miRNAs obtained by searching miRDB must be carefully examined via detailed experiments because these predictions may be derived from specific types of cancer cells that may have different responses to other types of cancer cells. This review sheds light on the connections between natural products, miRNAs, and AKT pathways and can provide a future direction for exploring the regulation of cancer cell functions by natural products.

## Figures and Tables

**Table 4:** Connecting natural product-affected miRNAs to predict targeted AKT and AKT effectors.

	AKT1/2/3			AKT Effectors								AKT1/2/3				AKT Effectors					
	AKT1	AKT2	AKT3	FOXO1	FOXO3	MYC	MTOR	AKT1S1	DEPTOR	HIF1A		AKT1	AKT2	AKT3	FOXO1	FOXO3	FOXO4	MYC	MTOR	DEPTOR	HIF1A
**let-7f-1-3p**				FOXO1							**miR-223-3p**				FOXO1	FOXO3					
**miR-101-3p**			AKT3				mTOR				**miR-22-3p**			AKT3							
**miR-103a-3p**		AKT2									**miR-23b-3p**					FOXO3	FOXO4				
**miR-106a-5p**			AKT3								**miR-23c**					FOXO3	FOXO4				
**miR-106b-5p**										HIF1A	**miR-24-3p**						FOXO4				
**miR-107**		AKT2									**miR-27a-3p**				FOXO1	FOXO3					
**miR-122-5p**			AKT3		FOXO3						**miR-27b-3p**				FOXO1	FOXO3					
**miR-124-3p**		AKT2	AKT3					AKT1S1	DEPTOR		**miR-29b-3p**		AKT2	AKT3		FOXO3	FOXO4				
**miR-125a-5p**								AKT1S1			**miR-29c-3p**		AKT2	AKT3		FOXO3	FOXO4				
**miR-125b-5p**								AKT1S1			**miR-302a-5p**										HIF1A
**miR-129-1-3p**								AKT1S1			**miR-324-5p**				FOXO1						
**miR-1303**					FOXO3						**miR-381-3p**			AKT3		FOXO3				DEPTOR	
**miR-133b**					FOXO3						**miR-384**					FOXO3					
**miR-143-3p**				FOXO1							**miR-421**					FOXO3			MTOR		
**miR-144-3p**				FOXO1			MTOR				**miR-424-5p**			AKT3						DEPTOR	
**miR-145-5p**			AKT3	FOXO1							**miR-4262**			AKT3						DEPTOR	
**miR-148a-3p**		AKT2									**miR-4284**					FOXO3					
**miR-151a-3p**			AKT3							HIF1A	**miR-486-5p**				FOXO1						
**miR-15a-5p**			AKT3								**miR-503-5p**			AKT3							
**miR-15b-5p**			AKT3						DEPTOR		**miR-506-3p**			AKT3						DEPTOR	
**miR-16-2**						MYC				HIF1A	**miR-513a-3p**				FOXO1						
**miR-16-5p**			AKT3						DEPTOR		**miR-518c-5p**			AKT3							
**miR-17-5p**			AKT3							HIF1A	**miR-518f-5p**			AKT3		FOXO3					
**miR-181a-5p**			AKT3						DEPTOR		**miR-5195-3p**			AKT3	FOXO1						
**miR-181b-5p**			AKT3						DEPTOR		**miR-520h**									DEPTOR	HIF1A
**miR-181c-5p**			AKT3						DEPTOR		**miR-545-3p**			AKT3					MTOR	DEPTOR	
**miR-182-5p**				FOXO1	FOXO3				DEPTOR		**miR-590-5p**					FOXO3					
**miR-183-5p**				FOXO1							**miR-608**						FOXO4				
**miR-186-5p**			AKT3							HIF1A	**miR-616-3p**								MTOR		
**miR-18a-5p**										HIF1A	**miR-622**										HIF1A
**miR-18b-5p**										HIF1A	**miR-629-5p**			AKT3				MYC			
**miR-195-5p**			AKT3						DEPTOR		**miR-7-1-3p**	AKT1			FOXO1						HIF1A
**miR-199a-5p**										HIF1A	**miR-7-5p**			AKT3							
**miR-203a-3p**						MYC				HIF1A	**miR-93-5p**			AKT3							HIF1A
**miR-20a-5p**			AKT3							HIF1A	**miR-9-5p**					FOXO3					
**miR-21-5p**					FOXO3						**miR-96-5p**				FOXO1				MTOR	DEPTOR	
**miR-22-3P**			AKT3								**miR-99a-5p**								MTOR		

The above natural product-affected miRNAs were derived from Table 3. Some AKT effectors are not listed because they could not be retrieved using the miRDB database (date: 11 November 2022).
